# Cysteine Leukotriene Receptor Antagonist-Montelukast Effects on Diabetic Retinal Microvascular Endothelial Cells Curtail Autophagy

**DOI:** 10.1167/iovs.65.13.15

**Published:** 2024-11-06

**Authors:** Ahmed M. Awad, Amritha T. M. Seetharaman, Mohammad Shahadat Hossain, Sally L. Elshaer, Rania R. Abdelaziz, Manar A. Nader, Rajashekhar Gangaraju

**Affiliations:** 1 Department of Pharmacology and Toxicology, Faculty of Pharmacy, Mansoura University, Mansoura, Egypt; 2 Department of Ophthalmology, University of Tennessee Health Science Center, Memphis, Tennessee, United States; 3 Department of Anatomy & Neurobiology, University of Tennessee Health Science Center, Memphis, Tennessee, United States; 4 Department of Pharmacology and Toxicology, Faculty of Pharmacy, Mansoura National University, Gamasa, Mansoura, Egypt

**Keywords:** leukostasis, vascular permeability, TNF-α, diabetes, inflammation, beclin-1, p62/sequestosome, VE-cadherin, diabetic retinopathy, diabetic macular edema, CysLTR1, leukotrienes

## Abstract

**Purpose:**

Diabetic macular edema (DME) is the primary cause of vision impairment in diabetic retinopathy (DR) patients. A previous study has shown the efficacy of montelukast, a cysteinyl leukotriene receptor (CysLTR)1 antagonist, in a diabetic mouse model. This study aims to understand the CysLTR1 signaling in retinal endothelial cells and the impact of montelukast.

**Methods:**

Primary human retinal microvascular endothelial cells (HRECs) challenged with 20 ng/mL TNF-α and 30 mM D-glucose (D-glu) for six to 24 hours served as a model of endothelial activation. HRECs were incubated with L-glucose (L-glu) as an osmotic control. CysLTR1 knockdown and montelukast pretreatment assessed CysLTR1 antagonism. Gene expression, protein expression, and cell-permeable dyes were utilized to measure autophagy and inflammation. Transendothelial electrical resistance (TER) and transendothelial migration of mononuclear leukocytes across HRECs monolayer were measured as a functional assessment of vascular permeability.

**Results:**

Endothelial activation induced by hyperglycemia and inflammation increased CysLTR1 expression, triggering autophagy within two to six hours, IL-1β production, loss of junction integrity, decreased TER, and increased leukocyte migration within six to 24 hours. Pretreatment with montelukast effectively alleviated these effects, demonstrating its dependence on CysLTR1.

**Conclusions:**

Dysfunctional retinal endothelium initiates a self-reinforcing loop of inflammation, autophagy, and compromised integrity associated with heightened CysLTR1 levels. The antagonistic effect of montelukast against CysLTR1 effectively mitigates these detrimental changes. This study reveals CysLTR1 as a potential therapeutic target in treating DME and offers a novel strategy to mitigate detrimental changes in DR.

Diabetes gives rise to complications affecting various organs, and one significant microvascular complication is diabetic retinopathy (DR).^1^ The global incidence of DR is projected to reach 700 million in 2045, with Middle Eastern and North American countries affected disproportionately.^2^ Among individuals with type 2 diabetes in the United States, approximately 40.3% are estimated to have DR, with 8.2% experiencing retinopathy that poses a threat to vision.^3^ In the case of type 1 diabetes patients, 86% have retinopathy, and 42% exhibit vision-threatening stages of DR.^4^ DR develops because of prolonged damage inflicted by hyperglycemia on the microvessels within the retina.^5^ This condition stands as a leading cause of blindness among those in the working-age population.

DR has long been recognized as a microvascular ailment, as evident from the damage to vascular endothelial cells and the breakdown of the blood-retinal barrier observed in the early stages of the condition.^6^ Notably, diabetic macular edema (DME), characterized by abnormal intraretinal fluid accumulation in the macular region, stands out as the primary cause of vision impairment in individuals with DR.^7^ Indeed, the terms DR and DME are not interchangeable. Clinically, DME could occur at any stage of DR, the latter classified by vascular lesions. Therefore, the pathophysiology and risk factors for DME may be in part distinct from the pathophysiology of DR and its neurovascular changes in the retina. Both experimental and clinical studies have underscored the involvement of inflammation in DME.^8^ Our prior investigations have proposed that inflammation and hyperglycemia-induced endothelial activation led to endoplasmic reticulum (ER) stress–mediated changes in intercellular junctions and increased transmigration of leukocytes, thereby contributing to vascular permeability and tissue inflammation in diabetic mice.^9^^,^^10^ Recent literature indicates that ER stress might exert widespread effects on cytokine-induced inflammatory responses and autophagy.^11^^,^^12^ Autophagy is a highly conserved catabolic pathway employed by cells to eliminate misfolded or aggregated proteins and damaged organelles.^13^ Despite the existing studies, the question of whether autophagy hinders or promotes the progression of DME or DR remains uncertain.^14^

Leukotrienes (LTs), bioactive molecules derived from arachidonic acid, mediate allergic and inflammatory reactions.^15^ Although leukotrienes play a role in the body's defense against foreign entities or microorganisms, excessive production, as seen in diabetes, can lead to various immune and inflammatory complications. The synthesis of LTs involves a series of enzymatic reactions resulting in the production of LTA4, LTB4, LTC4, LTD4, and LTE4, collectively known as cysteinyl leukotrienes (CysLTs). Although LTB4 is well known for its role as a leukocyte attractant and has been extensively studied,^16^^,^^17^ recent attention has focused on LTC4, LTD4, and LTE4 leukotrienes, along with their corresponding receptors known as Cysteinyl leukotriene receptors (CysLTR).^18^

Montelukast, an antagonist approved by the Food and Drug Administration for targeting CysLTR1, is used to manage various inflammatory conditions, including asthma and rheumatoid arthritis.^19^ Recent findings suggest that montelukast effectively prevents diabetes-induced retinal capillary and neuronal degeneration in a streptozotocin-induced diabetes mouse model.^20^ Although CysLTRs have been extensively investigated in immune cells, their specific impact on the molecular pathways in retinal vascular endothelium initiating autophagy, inflammation, and microvascular permeability as a measure of DME has not been thoroughly explored. Therefore this study aims to understand the CysLTR1 signaling in retinal endothelial cells using a well-established model of endothelial activation induced by hyperglycemia combined with proinflammatory signaling. Our study demonstrates the heightened expression of CysLTR1 in activated retinal endothelial cells with increasing time. This increased expression is associated with elevated autophagy, creating a self-reinforcing loop of inflammation, leading to compromised junction integrity and increased permeability. The antagonistic effect of montelukast against CysLTR1 effectively mitigates these detrimental changes.

## Material and Methods

### Cell Culture and Experimental Setup

Primary human retinal microvascular endothelial cells (HRECs; Cat. no. ACBRI 181; Cell Systems, Inc., Kirkland, WA, USA) were routinely cultured in EGM-2MV medium (Cat. no. CC-4147; Lonza Group, Basel, Switzerland) in collagen coated 10 cm dishes (113 µL of 3 mg/mL in 20 mM glacial acetic acid, Cat. no. A10483-01; Life Technologies, Carlsbad, CA, USA) and incubated in a humidified chamber set at 37°C and 5% CO_2_. After reaching 60% to 80% confluency, cells were seeded at 1 × 10^5^ cells per well of 12-well collagen-coated plates (7 µL of 3 mg/mL collagen per well). After reaching 60% to 80% confluency, cells were shifted into serum-free medium (EBM-2, Cat. no. CC-3156; Lonza Group) overnight. After this, cells receiving montelukast were pretreated with Montelukast Sodium (Mon; Cat. no. 151767-02-1; Millipore Sigma, Burlington, MA, USA; 5-20 µM) for 30 minutes and challenged with 20 ng/mL TNF-α (Cat. no. 10602 HNAE; Sino Biological US, Inc., Wayne, PA, USA) and 30 mM D-Glucose (D-glu; Cat. no. G7528; Millipore Sigma) for six to 24 hours. HRECs incubated with 30 mM L-Glucose (L-glu; Cat. no. 241920010; Thermo Fisher Scientific, Waltham, MA, USA), an enantiomer of the D-glucose, served as an osmotic control. The cells were then used in subsequent experiments described below.

### Assessment of Autophagosomes

To assess autophagic vacuoles (autophagosomes), CYTO-ID Autophagy detection kit (Cat. no. ENZ-51031; Enzo Life Sciences, Farmingdale, NY, USA) was used by selectively labeling accumulated autophagic vacuoles in lysosomal inhibited live cells. Briefly, HRECs were grown on 24-well plates containing coverslips until they reached a 50% to 70% level of confluence. The cells were incubated with various treatments. Rapamycin, a known inducer of autophagy, served as positive control. After the respective treatments, the medium was removed, and the cells were washed twice with 1X assay buffer containing 2% FBS. After this, 200 µL of microscopy dual detection reagent (1 mL of 1X assay buffer, 5% FBS, 2 µL of CYTO-ID Green Detection Reagent [Enzo Life Sciences], and 1 µL of Hoechst 33342 Nuclear Stain [Thermo Fisher Scientific]) were then dispensed to cover each sample of monolayer cells. Cells were then protected from light and incubated for 30 minutes at 37°C. After this, cells were carefully washed with 1X assay buffer. Fixation was then done by incubation for 20 minutes with 4% formaldehyde. After this, cells were washed three times with 1X assay buffer. Coverslips were placed on microscope slides and analyzed by confocal microscopy.

### RNA Extraction and Reverse Transcription

At the end of the study, cells were lysed with 300 µL Lysis buffer (Cat. no. 593-84-0; Macherey-Nagel GmbH & Co KG, Düren, Germany), and RNA was isolated as per the manufacture instructions, using NucleoSpin miRNA kit (Cat. no. 740971.250; Macherey-Nagel GmbH & Co KG). Equal amount of RNA (250 ng) was used in reverse transcription using multiscribe reverse transcriptase (Cat. no. 4319983), dNTP mix (Cat. no. 4367381), 10x RT random primers (Cat. no. 4319979), and 10x RT buffer (Cat. no. 4319981), Thermo Fisher Scientific, yielding 20 µL of cDNA per reaction. The concentration of cDNA was measured using the NanoDrop ND-1000 Spectrophotometer (Thermo Fisher Scientific) and diluted with nuclease-free water to be 100 ng/µL for the polymerase chain reaction (PCR).

### PCR

For each reaction, 5 µL PowerUp SYBR Green Master Mix (Cat. no. A25742; Thermo Fisher Scientific), 1 µL of both forward and reverse primers (10 µM), 1 µL nuclease-free water, and 2 µL of cDNA were used to measure gene expression of beclin-1, p62/SQSTM1, and IL-1β (Table). EF‐1α served as a housekeeping internal control. The cDNA samples were also used for real-time quantitative PCR to study the expression levels of CCL-2, IL-6, and VCAM-1 using TaqMan assays (Table). The 18S rRNA served as a housekeeping internal control. In both assays, the expression levels of target gene transcripts were determined using 2^−ΔΔCt^ method and normalized to the housekeeping gene control.

**Table.** Taqman Probes and Sybr Green PCR Assay Primers

Genes	Taqman Assay ID/Primer Sequence		Catalog No. or Assay ID	Company
Beclin-1	Forward	5-GGG CTC CCG AGG GAT GG-3	434577760	Integrated DNA Technologies (IDT)
	Reverse	5-CTC GTG TCC AGT TTC AGG GG-3	434577761	
P62/SQSTM1	Forward	5-GCC TGA GGC GGA AGC C-3	434577762	IDT
	Reverse	5-CCC GTC CTC ATC GCG GTA-3	434577763	
IL-1β	Forward	5-TTC GAG GCA CAA GGC ACA A-3	411163999	IDT
	Reverse	5-TGG CTG CTT CAG ACA CTT GAG-3	411163998	
EF-1α	Forward	5-AGA TTG ATC GCC GTT CTG GT-3	409722451	IDT
	Reverse	5-AGC AAA GCG ACC CAA AGG T-3	409722452	
Chemokine (C-C motif) chemokine ligand 2 (CCL-2)			Hs00234140_m1	Thermo Scientific
Vascular cell adhesion molecule 1(VCAM-1)			Hs01003372_m1	Thermo Scientific
IL-6			Hs00174131_m1	Thermo Scientific
18S			Hs03003631_g1	Thermo Scientific

### Immunocytochemistry

In a 24-well plate, endothelial cells were cultured on 10 mm circular collagen-coated coverslips until the required confluency was reached. After the necessary treatments, the cells were fixed for 15 minutes with 4% paraformaldehyde and blocked for an hour with 5% BSA in 0.1% triton × 100. After this the cells were incubated with primary antibodies against CysLTR1 (Cat. no. PA5-97651 [Invitrogen, Carlsbad, CA, USA] or Cat. no. Ab151484 [Abcam, Cambridge, MA, USA]), p62/Sequestosome (SQSTM)1 (Cat. no. ab109012; Abcam), and VE-cadherin (Cat. no. sc-9989, F-8 mouse monoclonal; Santa Cruz Biotechnology, Dallas, TX, USA) for overnight at 4°C. This was followed by washing and incubation with the corresponding secondary antibody. For nuclear localization, cells were incubated with DAPI for five minutes. Stained cells on coverslips were mounted using an aqueous mounting medium (Cat. no. P36961, Prolong Diamond Anti-fade mountant; Invitrogen) and were visualized using a Zeiss LSM 900 laser scanning confocal microscope (Zeiss, Oberkochen, Germany).

### Western and Slot-Bot Analysis

Cells were lysed by RIPA Lysis buffer (Cat. no. 89900; Thermo Fisher Scientific). The protein concentration was evaluated using BCA protein assay kit (Cat. no. 23227; Thermo Fisher Scientific). The samples containing 15 µg protein were mixed with 2x Laemmli buffer (Cat. no. S3401-1VL; Sigma-Aldrich Corp., St. Louis, MO, USA) and heated for 10 minutes at 70°C for protein denaturation. Protein samples were separated using SDS-PAGE gel electrophoresis, then blotted into nitrocellulose membrane. In some experiments, about 10 µg protein was analyzed using the Slot blot plate method (Cat. no. 170-6542, Bio-slot SF; Bio-Rad Life Science, Hercules, CA, USA) according to manufacturer instructions. The membrane was blocked for one hour at room temperature in 3% BSA and incubated overnight at 4°C with the primary antibody for CysLTR1 (Cat. no. PA5-97651; Invitrogen) and p62/SQSTM1 (Cat. no. AB109012; Abcam), and β-tubulin (Cat. no. MAB5564; EMD Millipore, Burlington, MA, USA) as a housekeeping protein control. The membrane was then washed with TBST and incubated with anti-rabbit HRP-conjugated secondary antibody (Cat. no. 7074S; Cell Signaling Technology, Danvers, MA, USA) for two hours at room temperature. After washing, the chemiluminescent signal was developed by the chemiluminescent substrate (Cat. no. 34580; Thermo Fisher Scientific) and the images were captured using Odyssey Fc imager (LICOR Odyssey Fc Model-2800; LI-COR Biosciences, Lincoln, NE, USA). Image J software (National Institutes of Health) was used to read the band intensity of the target proteins against β-tubulin housekeeping protein control.

### In Vitro Assessment of Retinal Endothelial Cell Permeability

An electric cell-substrate impedance sensing device (ECIS Zθ; Applied Biophysics, Inc., Troy, NY, USA) was used to measure trans-endothelial electrical resistance (TER), as previously reported.^9^ Briefly, HRECs were seeded on gold electrodes (8W10E+; Applied Biophysics, Inc.) at a density of 2 × 10^5^ cells/mL in a 400 µL volume per well. Following a 16-hour growth period to reach their maximum resistance of approximately 1200 Ω, the cells were subjected to several treatments, and any alterations in resistance were observed for 24 hours.

### Knockdown of CysLTR1

HRECs at 60% to 80% confluence were used in the assay. Lipofectamine 2000 transfection reagent (Cat. no. 11668019; Life Technologies, Thermo Fisher Scientific) and CysLTR1 siRNA (Cat. no. 439240; Ambion, Austin, TX, USA) were diluted in OptiMEM (Cat. # 11058021; Gibco, Thermo Fisher Scientific) media, mixed and incubated for five minutes. The mixture was added to the cells and cultured for 24 hours. Cells incubated with control siRNA (Cat. no. 4390844; Ambion) served as negative controls. The cells were then used in subsequent experiments with various treatments.

### Transendothelial Migration Assay

Transendothelial migration of mononuclear leukocytes across the monolayer of HRECs was performed as described previously.^21^ Briefly, HRECs were cultured in collagen coated transwell filter inserts (Cat. no. 25-289, Gen-clone; Genesee Scientific, Morrisville, NC, USA) for two days. After specific treatments, the inserts were placed in fresh wells with serum free medium, and 1 × 10^6^ fluorescently labeled monocyte cells were added to the upper compartment. After 24 hours, the number of transmigrated leukocytes was measured in the media from the bottom well by measuring the fluorescence intensity at an excitation of 549 nm and emission at 565 nm. Representative images of the transmigrated leukocytes were captured using EVOS fluorescence microscope (Thermo Fisher Scientific).

### Statistical Analysis

Statistical analysis was conducted using one-way analysis of variance (ANOVA) followed by the Tukey-Kramer post-hoc test. Data were expressed as mean ± SEM. GraphPad Prism Version 10 was used for statistical analysis, and a *P* value < 0.05 was considered significant.

## Results

### Effect of Hyperglycemia and TNF-α–Induced Endothelial Activation on Autophagy and Inflammation

**Figure 1.**
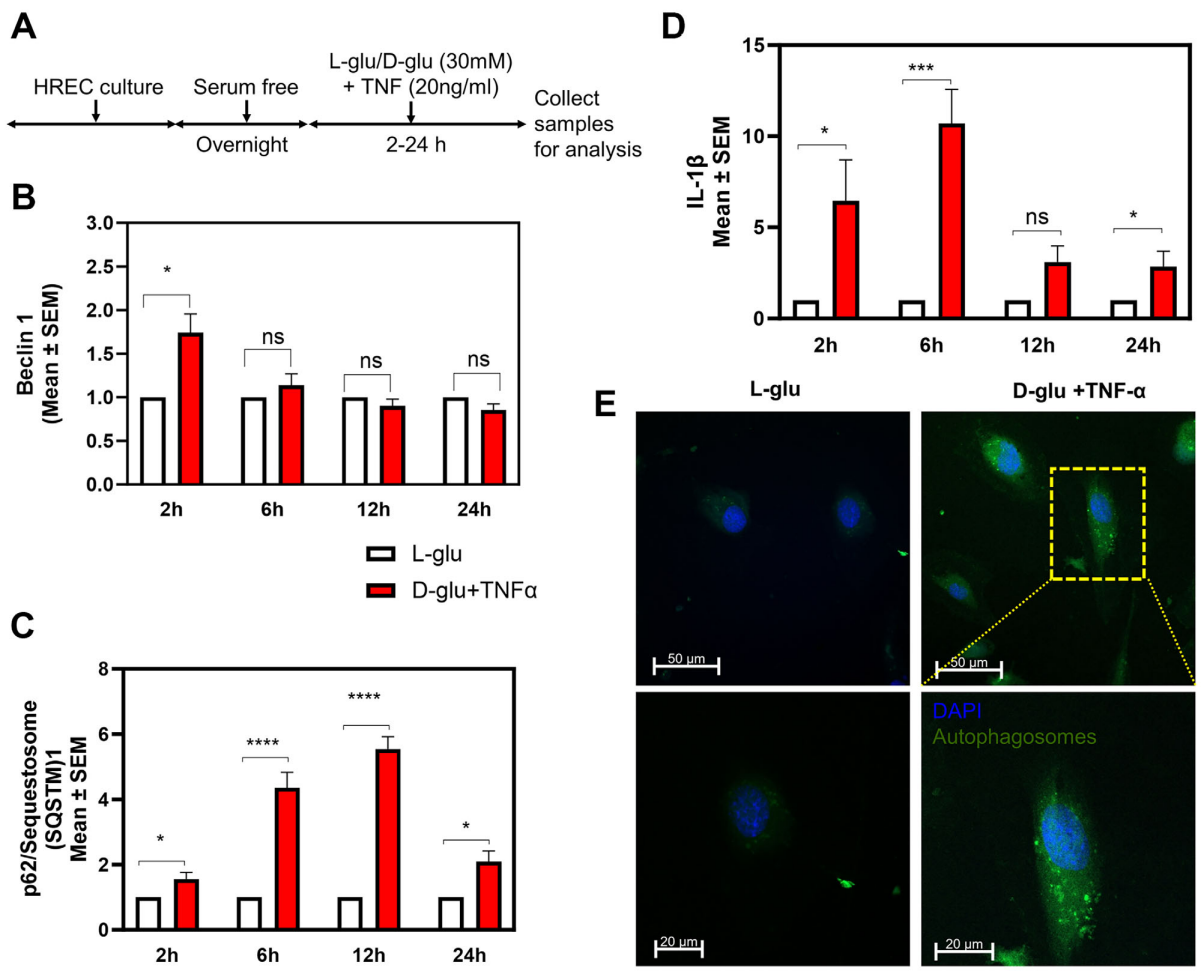
Effect of hyperglycemia and TNF-α-induced endothelial activation on autophagy and inflammation. (**A**) Timeline of HREC culture and analysis. Gene expression of beclin-1 (**B**), p62/SQSTM1 (**C**), and IL-1β (**D**) at different time points after challenging with hyperglycemia and TNF-α. (**E**) Autophagic vacuoles (autophagosomes) were assessed six hours after treatment with hyperglycemia and TNF-α. *P < 0.05; **P < 0.01; ***P < 0.001, ****P < 0.0001; ns, not significant.

Our previous studies using HRECs exposed to high glucose (30 mM, D-glu) and TNF-α (20 ng/mL) elicited robust ER stress with an additive effect compared to a single agent alone.^10^ In this study we first determined whether HRECs exposed to high glucose and TNF-α elicit autophagic response (Fig. 1A). HRECs treated with D-glu + TNF-α resulted in a significant increase (*P* < 0.05) in beclin-1, an early autophagy marker compared to L-glu within two hours with later time points up to 24 hours have no influence on its expression (Fig. 1B). On the other hand, D-glu + TNF-α treatment resulted in a significant increase in p62/SQSTM1 compared to L-glu at all time points with the highest increase (*P* < 0.001) noted at six and 12 hours (Fig. 1C). Furthermore, D-glu + TNF-α treatment resulted in a significant increase (*P* < 0.05, 0.001, 0.05) in IL-1β expression compared to L-glu at two, six, and 24 hours, respectively (Fig. 1D). Additional studies using 5 mM D-glu, 30 mM D-glu or L-glu + TNF-α confirmed specific synergistic increases in these autophagic markers with D-glu + TNF-α treatment (Supplementary Fig. S1A). Based on this initial study, we concluded that two hours is a suitable time point for detection of early autophagy, whereas six hours is the most reliable time point for detection of late-stage autophagy and inflammation. To further validate the influence of D-glu + TNF-α on HRECs to induce autophagy, autophagic vacuoles (autophagosomes) were shown to increase by selectively labeling accumulated autophagic vacuoles in lysosomal inhibited live cells compared to those cells treated with L-glu (Fig. 1E).

### Effect of Montelukast on the Hyperglycemia and TNF-α–Induced Endothelial Activation on Autophagy

**Figure 2.**
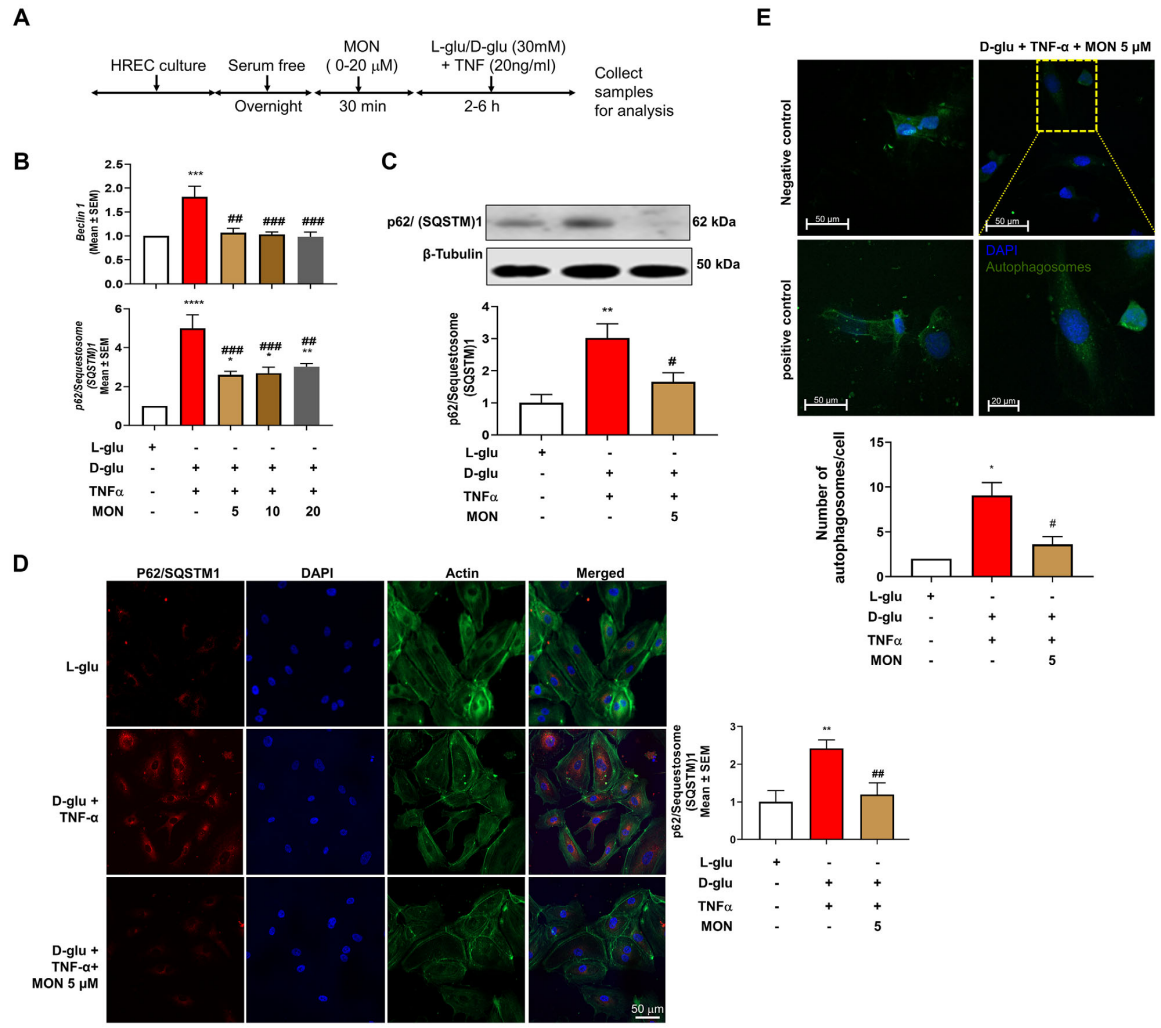
Effect of montelukast on the hyperglycemia and TNF-α–induced endothelial activation on autophagy. (**A**) Timeline of HREC culture and analysis with and without montelukast (*Mon*). (**B**) Gene expression of beclin-1 and p62/SQSTM1. (**C**) Western blot protein expression of p62/SQSTM1 at different time points after challenging with hyperglycemia and TNF-α. (**D**) Representative photomicrographs of various treatments to assess p62/SQSTM1 protein by immunocytochemistry and quantification. (**E**) Autophagic vacuoles (autophagosomes) were assessed six hours after treatment with hyperglycemia and TNF-α with and without pretreatment with montelukast. *P < 0.05; **P < 0.01; ***P < 0.001, ****P < 0.0001 compared to L-glu group. #*P* < 0.05; ##*P* < 0.01; ###*P* < 0.001 compared to D-glu + TNF-α group.

Next, we asked if montelukast could ameliorate the D-glu + TNF-α associated changes in autophagy (Fig. 2A and Supplementary Fig. S1A). To this end, HRECs pretreated with montelukast and exposed to D-glu + TNF-α resulted in a significant decrease (*P* < 0.01) in beclin-1 (Fig. 2B) and p62/SQSTM1 gene expression (Fig. 2B) at all doses tested (5, 10, and 20 µM) with no dose-dependent relationship. Because there was no significant difference among the three concentrations of montelukast studied here, we only used 5 µM in the subsequent experiments.

Additional studies with immunocytochemistry and western blotting demonstrated nearly 3-fold increase in p62/SQSTM1 protein levels in HRECs exposed to D-glu + TNF-α (*P* < 0.01) with a significant (*P* < 0.01) decrease in HRECs pretreated with montelukast (5 µM) (Fig. 2C). Although HRECs exposed to L-glu + TNF-α increased p62/SQSTM1 protein levels substantially, a further significant increase was noted with D-glu + TNF-α (Supplementary Fig. S1B). Interestingly, regardless of the stimulating agent, montelukast reduced p62/SQSTM1 protein levels. Notably, there was no significant difference between montelukast (5 µM) and L-glu group (Fig. 2C and Supplementary Fig. S1B). Additionally, as shown by representative immunocytochemistry images, HRECs challenged with D-glu + TNF-α resulted in a significant increase (*P* < 0.01) in p62/SQSTM1 expression compared to L-glu group. On the other hand, HRECs pretreated with montelukast (5 µM) significantly decreased (*P* < 0.01) p62/SQSTM1 expression compared to D-glu + TNF-α group (Fig. 2D). Finally, pretreatment with montelukast (5 µM) significantly decreased (*P* < 0.05) the number of autophagosomes compared to those cells exposed to D-glu+TNF-α to comparable levels to that of L-glu group (Figs. 1E, 2E). Autophagy inducer, rapamycin and cells incubated with vehicle alone served as positive and negative controls, respectively confirmed the authenticity of the observed autophagosomes in the assay.

### Effect of Montelukast on the Hyperglycemia and TNF-α–Induced Endothelial Activation on Inflammation and Barrier Loss

**Figure 3.**
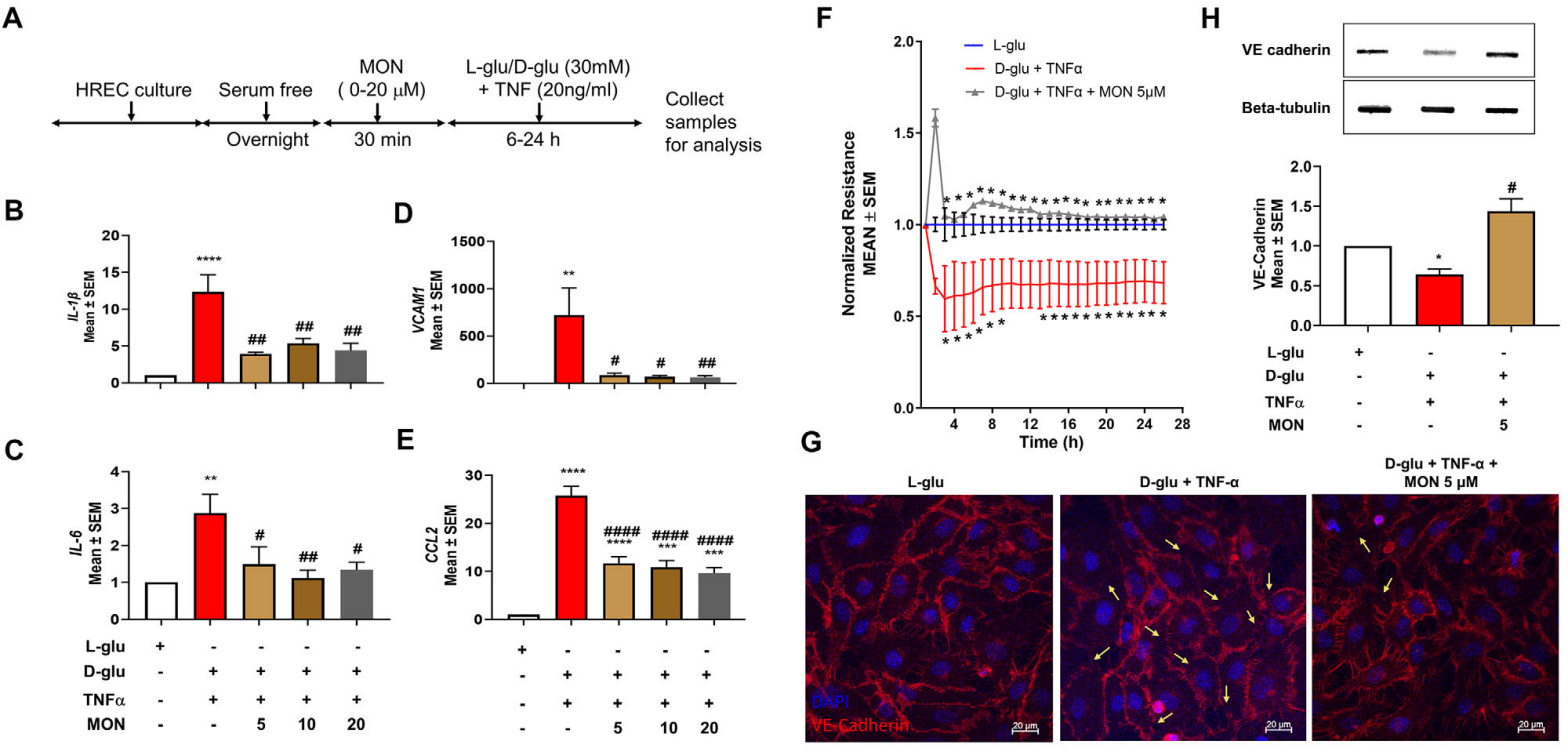
Effect of montelukast on inflammatory markers and barrier integrity in endothelial cells induced by hyperglycemia and TNF-α. (**A**) Timeline of HREC culture and analysis with and without montelukast (*Mon*). Effect of pretreatment with montelukast on IL-1β (**B**), IL6 (**C**), VCAM1 (**D**), and CCL2 (**E**) gene expression in HRECs challenged with hyperglycemia and TNF-α for six hours. (**F**) Effect of pretreatment with montelukast on barrier integrity (normalized resistance) in HRECs challenged with hyperglycemia and TNF-α. Loss of cell-to-cell contacts and VE-cadherin in HRECs challenged with hyperglycemia and TNF-α as evidenced by VE-cadherin immunocytochemistry (**G**) and slot blot analysis (**H**). *P < 0.05; **P < 0.01; ***P < 0.001, ****P < 0.0001. #*P <* 0.05; ##*P* < 0.01; ####*P* < 0.0001 compared to D-glu + TNF-α group.

Next, we tested if montelukast could ameliorate the D-glu + TNF-α associated changes in inflammatory markers (Fig. 3A). As expected, HRECs exposed to D-glu + TNF-α but pretreated with montelukast resulted in a significant decrease (*P* < 0.01) in the expression of pro-inflammatory IL-1β (Fig. 3B) and IL-6 expression (Fig. 3C). Additionally, pretreatment with montelukast also resulted on a significant decrease in VCAM1 (Fig. 3D, *P* < 0.05 with 5 and 10 µM and *P* < 0.01 with 20 µM montelukast) and CCL2 (Fig. 3E, *P* < 0.0001), well-known adhesion molecules involved in transmigration of leukocytes across endothelium.

Previously, we have shown that HRECs exposed to D-glu + TNF-α resulted in a sustained reduction in barrier integrity as evidenced by decreased TER.^10^ Based on this, we next determined if the observed decrease in TER could be rescued with montelukast. To this end, HRECs were exposed to D-glu + TNF-α with and without montelukast. HRECs exposed to D-glu + TNF-α induced a sustained reduction in barrier integrity as evidenced by decreased TER compared to L-glu treated HRECs (at 11 hours: D-glu + TNF-α, 0.67 ± 0.13; L-glu, 1.0 ± 0.3 AU, *P* < 0.05). On the other hand, these effects were rescued by pretreatment with montelukast (1.08 ± 0.00 A.U., *P* < 0.02), suggesting that endothelial permeability defects could be alleviated with montelukast (Fig. 3F). Based on our previous observation that alterations in TER under the influence of D-glu + TNF-α treated HRECs coincided with a change in VE-Cadherin distribution,^10^ we performed immunocytochemistry and slot blot experiments to assess the status of VE-Cadherin. Coincident with the loss of TER in HRECs exposed to D-glu + TNF-α, a marked loss of cell-cell contacts and a significant decrease in VE-cadherin was noted with a significant protection against such loss of VE-cadherin in cells pretreated with montelukast (Figs. 3G, 3H).

### Effect of Montelukast on the Hyperglycemia and TNF-α–Induced Endothelial Activation on CysLTR1 Levels

**Figure 4.**
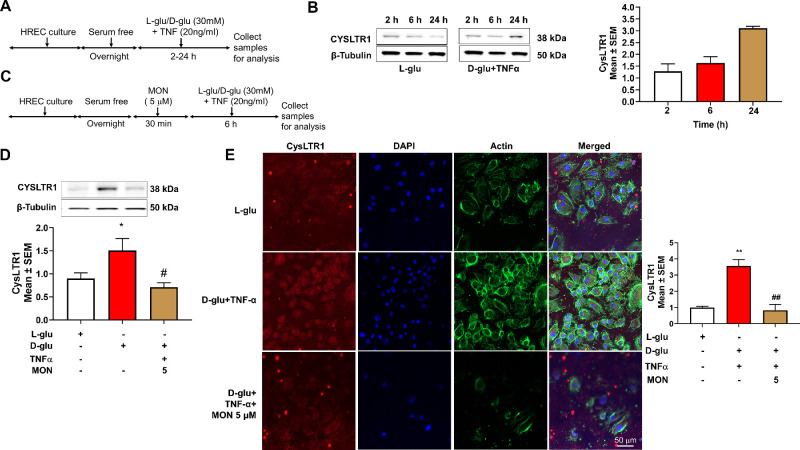
Effect of montelukast on the hyperglycemia and TNF-α–induced endothelial activation on CysLTR1 levels. (**A**) Timeline of HREC culture and analysis with hyperglycemia and TNF-α. (**B**) Western blot protein expression of CysLTR1 with increasing time. (**C**) Timeline of HREC culture and analysis with and without montelukast (*Mon*). Western blot protein expression of CysLTR1 with and without montelukast (**D**) immunocytochemistry and quantification (**E**) in HRECs challenged with hyperglycemia and TNF-α for six hours. *P < 0.05; **P < 0.01 compared to L-glu group. #*P* < 0.05; ##*P* < 0.01 compared to D-glu+ TNF-α group.

First, we established if D-glu + TNF-α alters the levels of CysLTR1 in HRECs (Fig. 4A). Although HRECs exposed to D-glu or TNF-α alone increased CysLTR1 protein levels marginally, a further significant increase was noted with D-glu + TNF-α (Supplementary Fig. S1B). With increasing time, the levels of CysLTR1 increased in HRECs when compared to L-glu group with six hours showing about a twofold increase (Fig. 4B). Because montelukast elicits its cell signaling via CysLTR1, we then tested whether montelukast alters the levels of CysLTR1 in HRECs (Fig. 4C). To this end, cell lysates from various treatments at six hours were assessed by Western blot for CysLTR1 protein levels. Although a significant twofold increase (*P* < 0.05) in CysLTR1 protein level was observed with HRECs exposed to D-glu + TNF-α compared to L-glu, a significant decrease in HRECs pretreated with montelukast 5 (µM) (*P* < 0.05) was observed compared to D-glu + TNF-α group (Fig. 4D). Interestingly, regardless of the stimulating agent montelukast reduced CysLTR1 protein levels. Notably, there was no significant difference between montelukast (5 µM) and L-glu group (Fig. 4D and Supplementary Fig. S1B). To confirm CysLTR1 in HRECs further, immunocytochemistry analysis revealed abundant expression of CysLTR1 in HRECs exposed to D-glu + TNF-α with a significant increase (*P* < 0.01) in CysLTR1 level compared to the L-glu group. On the other hand, HRECs pretreated with montelukast (5 µM) exposed to D-glu + TNF-α resulted in a significantly decreased (*P* < 0.01) CysLTR1 level (Fig. 4E) confirming the antagonistic effects of montelukast.

### CysLTR1 Knockdown Alleviates Montelukast Effects on Hyperglycemia and TNF-α–Induced Endothelial Activation on Autophagy and Barrier Loss

**Figure 5.**
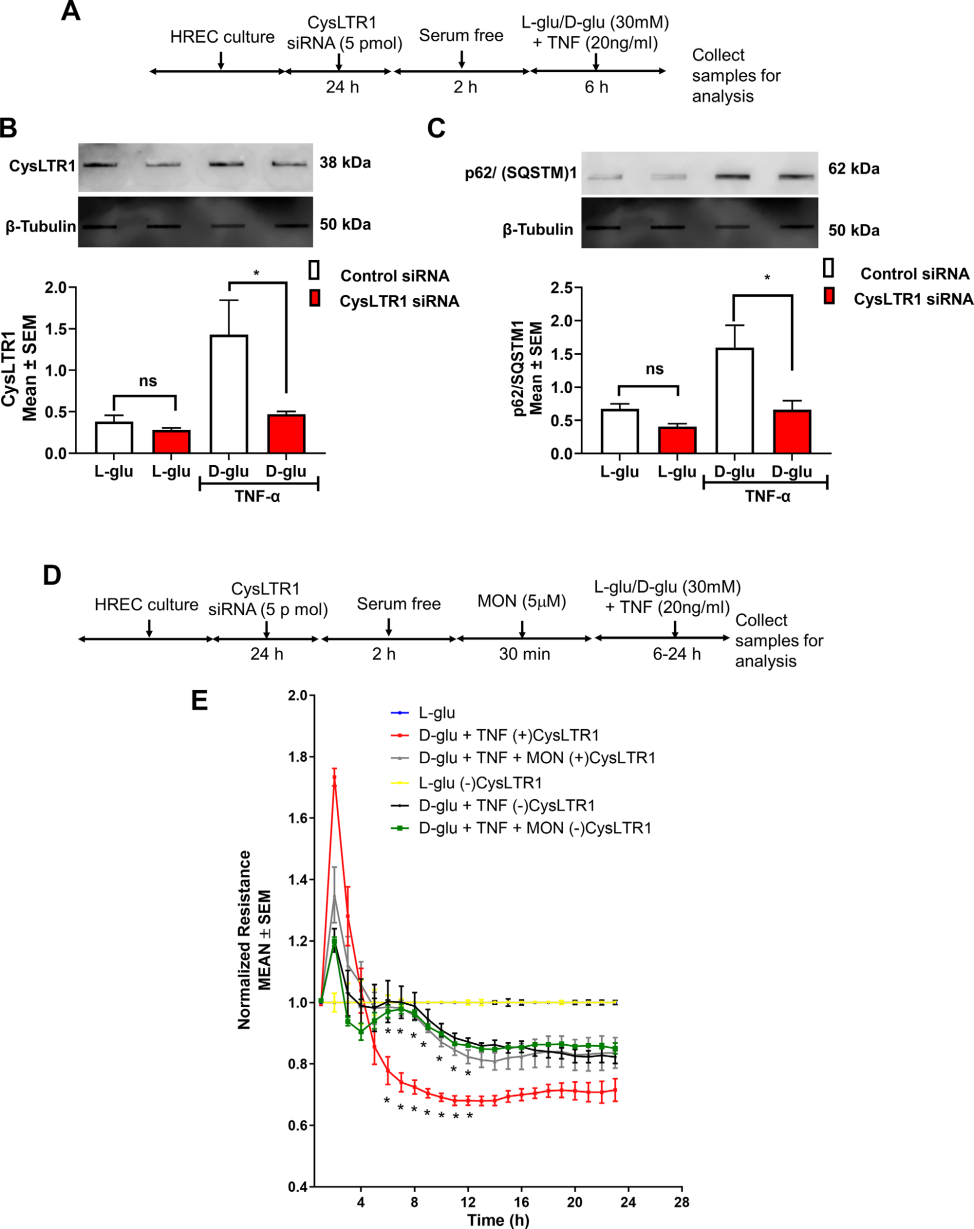
CysLTR1 knockdown alleviates montelukast's effects on hyperglycemia and TNF-α–induced endothelial activation on autophagy and barrier loss. (**A**) Timeline of CysLTR1 knockdown followed by treatment with hyperglycemia and TNF-α. Slot blot analysis of protein levels of CysLTR1 (**B**) and p62/SQSTM1 (**C**). (**D**) Timeline of CysLTR1 knockdown followed by pretreatment with montelukast and challenged with hyperglycemia and TNF-α. (**E**) Effect of pretreatment with montelukast on barrier integrity in HRECs challenged with hyperglycemia and TNF-α with (+) and without (−) CysLTR1 expression. *P < 0.05.

We performed a CysLTR1 knockdown experiment to determine the causal connection between CysLTR1 and montelukast in alleviating autophagy. To this end, we performed siRNA-mediated CysLTR1 knockdown in HRECs and challenged the cells with and without D-glu + TNF-α (Fig. 5A). As expected, D-glu + TNF-α treated cells and incubated with control siRNA demonstrated a robust increase in CysLTR1 levels with a significant decrease in HRECs incubated with CysLTR1 siRNA (*P* < 0.05) (Fig. 5B). In line with this observation, HRECs challenged with D-glu + TNF-α but incubated with CysLTR1 siRNA showed a remarkable decrease *(P* < 0.05) in p62/SQSTM1 levels compared to HRECs treated with D-glu + TNF-α group treated with control siRNA (Fig. 5C).

Next, we assessed the impact of CysLTR1 knockdown effects on endothelial permeability (Fig. 5D). Although HRECs incubated with control siRNA and exposed to D-glu + TNF-α induced a sustained reduction in barrier integrity beginning at six hours as evidenced by decreased TER compared to L-glu treated HRECs (at 10 hours: D-glu + TNF-α (+) CysLTR1, 0.69 ± 0.01; L-glu (+) CysLTR1, 1.0 ± 0.0 A.U., *P* < 0.001), these effects were rescued by pretreatment with Montelukast (at 10 hours: D-glu + TNF-α + Mon (+) CysLTR1, 0.84 ± 0.01 A.U., *P* < 0.001) (Fig. 5E). On the other hand, HRECs incubated with CysLTR1 siRNA and exposed to D-glu + TNF-α demonstrated a sustained reduction in barrier integrity, although with a significant difference compared to D-glu + TNF-α–treated HRECs incubated with control siRNA [at 11 hours: D-glu + TNF-α (−) CysLTR1, 0.88 ± 0.01; L-glu (−) CysLTR1, 1.0 ± 0.0 A.U., *P* < 0.01]. Interestingly, pretreatment with montelukast demonstrated a synergistic rescue in the D-glu + TNF-α–induced loss in TER in the absence of CysLTR1 [at 11 hours: D-glu + TNF-α + Mon (−) CysLTR1, 0.86 ± 0.00, *P* > 0.05] (Fig. 5E). This suggests that montelukast reduces endothelial permeability defects are indeed mediated through CysLTR1 in HRECs.

### CysLTR1 Knockdown Alleviates Montelukast Effects on Hyperglycemia and TNF-α–Induced Endothelial Activation on Inflammation and Barrier Loss

**Figure 6.**
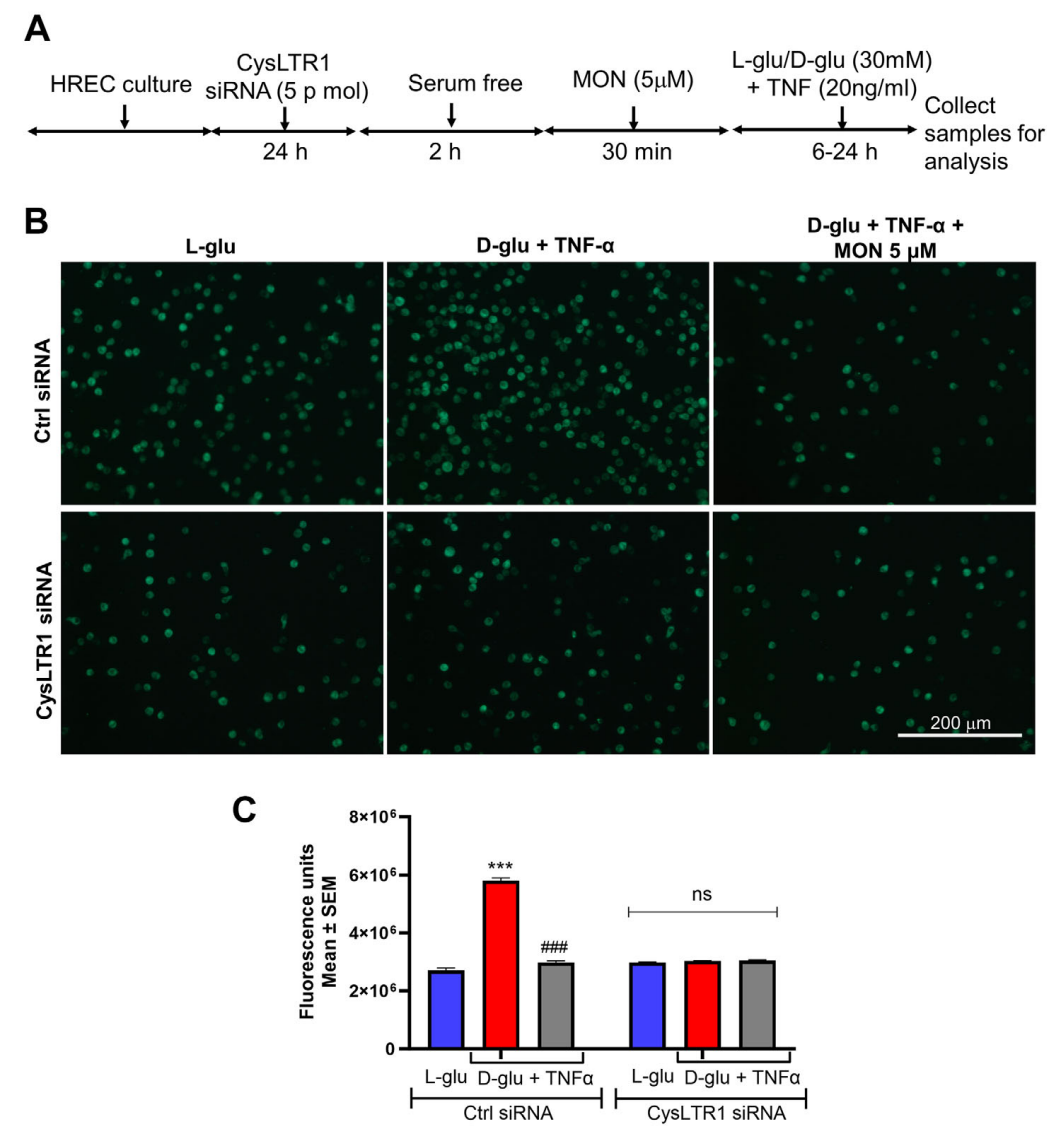
CysLTR1 knockdown alleviates montelukast's effects on hyperglycemia and TNF-α–induced endothelial activation on inflammation. (**A**) Timeline of CysLTR1 knockdown followed by pretreatment with montelukast and challenged with hyperglycemia and TNF-α. (**B**) Representative images of fluorescently labeled transmigrated leukocytes across HRECs with various treatments and quantification (**C**). ***P < 0.001 compared to L-glu group. ###*P* < 0.001 compared to D-glu + TNF-α group.

Previously, we have shown that transmigration of activated leukocytes across endothelial cells is a functional readout of compromised endothelium under the influence of inflammation.^22^ To this end we examined whether CysLTR1 is necessary for montelukast in alleviating inflammation under the influence of hyperglycemia and TNF-α. As described above, HRECs incubated with CysLTR1 siRNA and pretreated with and without montelukast and challenged the cells with and without D-glu + TNF-α to assess transendothelial migration of monocytes (Fig. 6A).

HRECs incubated with control siRNA and challenged with D-glu + TNF-α resulted in a significant increase in the monocytes transmigrated through endothelial cells (*P* < 0.001) compared to L-glu group (Figs. 6B, 6C). As expected, HRECs pretreated with montelukast (5 µM) and challenged with D-glu + TNF-α significantly decreased the number of transmigrated monocytes (*P* < 0.001) (Figs. 6B, 6C). On the other hand, those cells incubated with CysLTR1 siRNA and challenged with D-glu + TNF-α with and without montelukast demonstrated a significant reduction in transmigrated monocytes across the endothelium as compared to cells incubated with control siRNA. Interestingly, there was no difference in transmigrated monocytes among HRECs challenged with L-glu, D-glu + TNF-α, and montelukast (5 µM) (Figs. 6B, 6C), indicating that CysLTR1 has a central role in hyperglycemia and TNF-α–induced inflammation and endothelial activation, and montelukast associated receptor antagonism is necessary and sufficient to alleviate such effects.

## Discussion

This study investigated the role of CysLTR1 antagonism by montelukast in mitigating the detrimental effects of hyperglycemia and proinflammatory signaling on retinal microvascular endothelial cells (HRECs). The key findings of the study include (1) significant synergistic upregulation of CysLTR1 in HRECs challenged with hyperglycemia and TNF-α, suggesting its potential involvement in DME development; (2) upregulated CysLTR1 triggered autophagy within a short timeframe (two hours), followed by increased expression of proinflammatory IL-1β and endothelial adhesion proteins (six hours), which suggests a potential feed-forward loop contributing to DME progression; (3) pretreatment with montelukast effectively alleviated hyperglycemia and TNF-α–induced autophagy, inflammation, and loss of barrier function in HRECs. This protective effect depended entirely on CysLTR1 levels, highlighting its specific role in mediating these detrimental changes.

Research on autophagy in DR has increased significantly, however despite the studies that are now accessible, it is still unclear whether autophagy is promoting or inhibiting the progression of DR.^14^ The initial phase of autophagy is the initiation process, wherein beclin 1 plays a central role.^23^^,^^24^ After initiation, the elongation phase occurs, involving the interaction of LC3-II with p62/Sequestosome (SQSTM)1. This protein serves as a pivotal mediator of autophagy, possessing both a ubiquitin-binding domain and an LC3-interacting domain, facilitating the delivery of sequestered proteins to the autophagosomes.^25^ In line with these studies, HRECs challenged with hyperglycemia and TNF-α demonstrated increased beclin-1 within two hours and p62/Sequestosome at six hours, along with a marked increase in autophagosomes. These observations were alleviated when cells were pretreated with montelukast. Along these lines, others have shown activation of autophagy in the outer plexiform layer was linked to diabetes in C57BL/6J mice contributing to the loss of photoreceptors.^26^ In a different study, beclin-1 levels increased across the inner retina of STZ-induced diabetic mice when the mTOR pathway was suppressed. This upregulation was associated with signs of neuronal cell damage, such as the activation of apoptosis and a decrease in the number of cells in the ganglion cell layer.^27^ It is interesting to note that in diabetic mice, infusions of mTOR activator-MHY1485 blocked autophagy and significantly preserved neural cells.^27^ Similarly, in retinal pigment epithelial cells, it was shown that CysLTR1 can modulate autophagic activity.^28^^,^^29^ Recently, our group was able to show that montelukast can ameliorate the dysregulation in autophagy in chronic renal complications of diabetes,^30^ as well as aortic, and testicular complications.^31^

The synthesis of LTs involves a series of enzymatic reactions starting with the initiation by 5-lipoxygenase and 5-lipoxygenase–activating protein, resulting in the production of LTA4. LTA4 is rapidly converted to LTB4 by LTA4 hydrolase or alternatively to LTC4 by LTC4 synthase, a process requiring conjugation with glutathione. LTC4 can undergo further metabolism to produce LTD4 and LTE4, collectively forming the cysteinyl leukotrienes. Much literature has reported the effects of increased LTB4 in endothelial and epithelial cells and astrocytes with correlation to the development of DR.^16^^,^^17^^,^^32^^,^^33^ However, the role of other leukotrienes, including LTC4, LTD4, and LTE4, is unknown, although their corresponding receptors known as CysLTRs are recently shown to be predominantly expressed in ocular epithelial and neuronal tissues.^18^ To this end, our studies show for the first time that hyperglycemia and TNF-α–challenged HRECs demonstrate a synergistic increase in CysLTR1 levels with increasing exposure time. Moreover, its knockdown utilizing the siRNA approach or the use of montelukast completely abolishes endothelial dysfunction, as evidenced by altered TER and transendothelial migration of leukocytes. Although our studies did not attempt to study how hyperglycemia and TNF-α aid CysLTR1-associated changes in HRECs, it may be speculated that hyperglycemia and TNF-α–mediated stress might elevate LTE4, as also observed in preclinical models and diabetic subjects.^16^^,^^34^^,^^35^ However, a study in HRECs challenged with hyperglycemia failed to observe elevated LTE4^36^ leading to another speculation that the here observed effects may be independent of LTs and likely involve novel cell signaling pathways. Future research studying the direct impact of LTs on HRECs function and CysLTR1 expression and their comparison to high glucose and/or TNF-α may shed some light on this.

Recent findings suggest that montelukast effectively prevents DR by addressing retinal capillary and neurodegeneration in a mouse model^20^ and likely targets VEGF synthesis in diabetic retinal astrocytes.^32^ Additional studies have shown that montelukast preserved occludin levels in endothelial cells, potentially contributing to maintaining endothelial cell tight junctions and viability.^37^ Following these studies, our data show that pretreatment with montelukast effectively mitigates the loss of VE-cadherin, a prominent adheren junction protein involved in vascular permeability, and prevents permeability to activated leukocytes. Interestingly, montelukast effects synergized with siRNA knockdown studies to reduce CysLTR1 in alleviating endothelial barrier integrity and transmigration of leukocytes across the activated endothelium. The complete abolition of inflammation in the presence of both montelukast and siRNA targeting CysLTR1 suggests that the synergistic effect of these two approaches provides a more robust inhibition of the inflammatory pathway. Although montelukast blocks the receptor's activity, siRNA reduces the overall amount of CysLTR1, thereby preventing inflammation from being triggered even if CysLTR1 is present in low amounts.

We readily recognize some limitations of this study. (1) Although the study provides valuable insights, our studies are limited to in vitro endothelial culture; therefore further investigation using in vivo animal models of DME is crucial to confirm the findings and assess montelukast efficacy and safety in a more complex physiological setting. (2) Our study did not explore the precise mechanisms by which CysLTR1 activation influences autophagy and inflammation. Such studies could provide valuable insights for targeted therapeutic strategies. Similarly, investigating the potential synergistic effects of montelukast with other established DME treatment modalities could provide valuable insights into novel treatment strategies. (3) Although previous studies have shown that montelukast therapeutic benefits are possible as both a preventive and intervention strategy, our studies have only explored the pretreatment strategy, with future studies needing to explore whether similar beneficial effects are also possible when montelukast is given as an intervention after challenge with hyperglycemia and TNF-α. (4) Montelukast primarily acts through CysLTR1. However, blood vessels also express the CysLTR2,^20^^,^^38^ with its prominent role in retinal edema and ischemia-induced retinal pathogenesis^39^; it may be imperative to study the CysLTR1 and CysLTR2 receptor knockdowns in independent experiments, as well as in combination, which might better define the molecular pathways downstream of montelukast in DR.

In conclusion, this study demonstrates the potential of CysLTR1 antagonism by montelukast as a promising therapeutic approach for DME and potentially several ocular conditions.^40^ The evidence from this study and prior literature supports that montelukast may be beneficial for both DME and DR, suggesting that they share at least some common mechanistic pathways, but perhaps not all. By targeting the key CysLTR1 signaling pathway, montelukast may offer a novel strategy to mitigate detrimental changes in retinal endothelial cells, ultimately aiming to improve vision outcomes in diabetic patients. Further research is warranted to translate these promising findings into clinical applications for DME and DR management, with some early clinical trials already proving to be very effective in DR subjects^41^ with few other trials exploring diabetic kidney and cardiovascular diseases (NCT05498116 & NCT05362474, Clinicaltrials.org).

## Supplementary Material

Supplement 1

Supplement 2
