# Supplementary material for: Cysteine Leukotriene Receptor Antagonist-Montelukast Effects on Diabetic Retinal Microvascular Endothelial Cells Curtail Autophagy
Source: Invest Ophthalmol Vis Sci. 2024 Nov 6;65(13):15. doi: 10.1167/iovs.65.13.15 (PMC11549925; doi:10.1167/iovs.65.13.15)
Supplement: Supplement 1 [file iovs-65-13-15_s001.pdf]

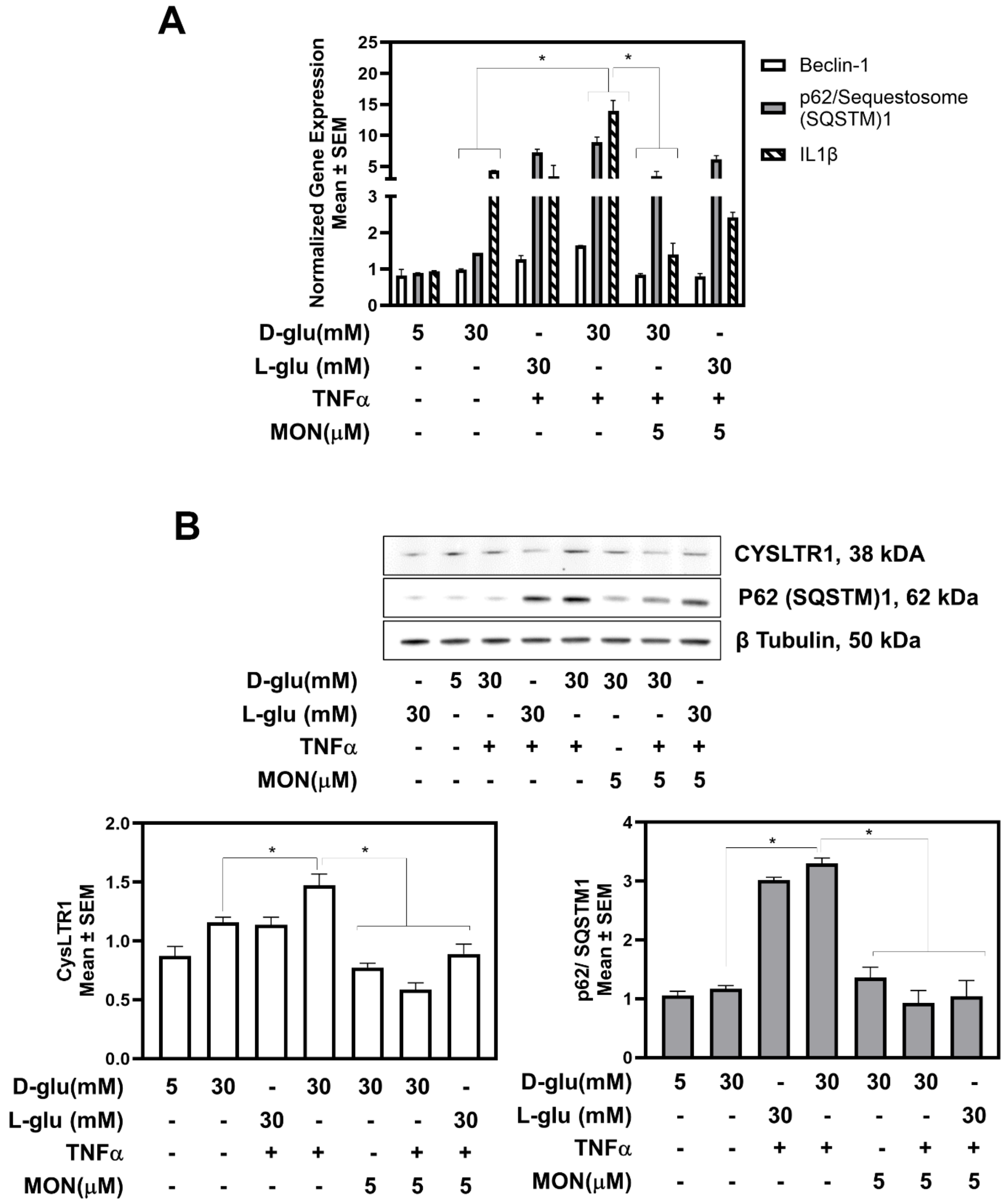

Supplemental Figure 1. Specific synergistic increase in autophagy and inflammation under hyperglycemia and TNF-α-induced stress in HRECs. (A). Gene expression of beclin-1, p62/SQSTM1, and IL-1β after challenging with various treatments. (B). Protein expression of CysLTR1 and p62/SQSTM1 after challenging with various treatments. *p<0.05.
