# Supplementary material for: Cysteine Leukotriene Receptor Antagonist-Montelukast Effects on Diabetic Retinal Microvascular Endothelial Cells Curtail Autophagy
Source: Invest Ophthalmol Vis Sci. 2024 Nov 6;65(13):15. doi: 10.1167/iovs.65.13.15 (PMC11549925; doi:10.1167/iovs.65.13.15)
Supplement: Supplement 2 [file iovs-65-13-15_s002.pdf]

Raw data

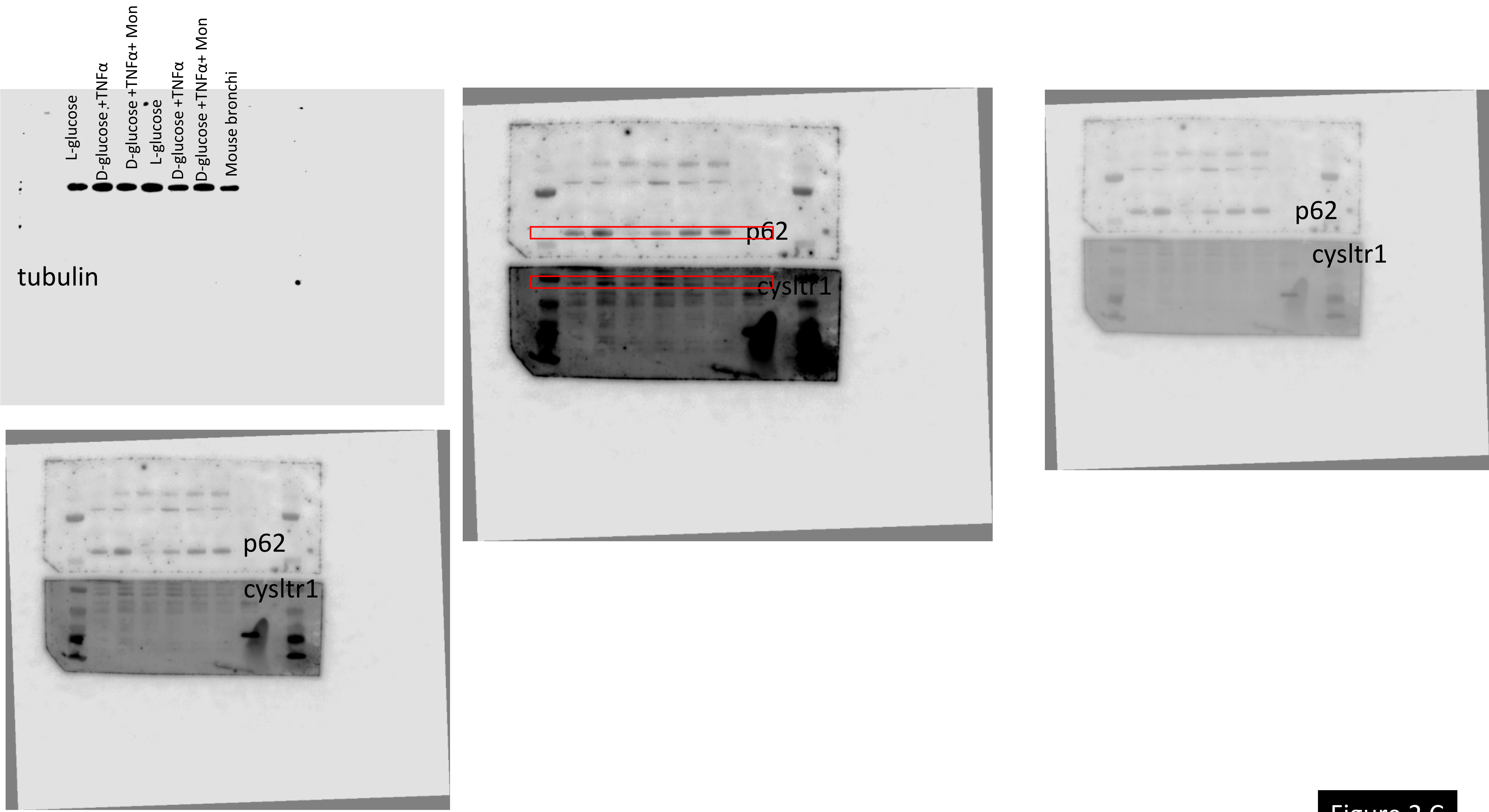
L-glucose
D-glucose +TNFα
D-glucose +TNFα+ Mon
L-glucose
D-glucose +TNFα
D-glucose +TNFα+ Mon
Mouse bronchi
tubulin
p62
cysltr1
p62
cysltr1
p62
cysltr1

Figure 2 C

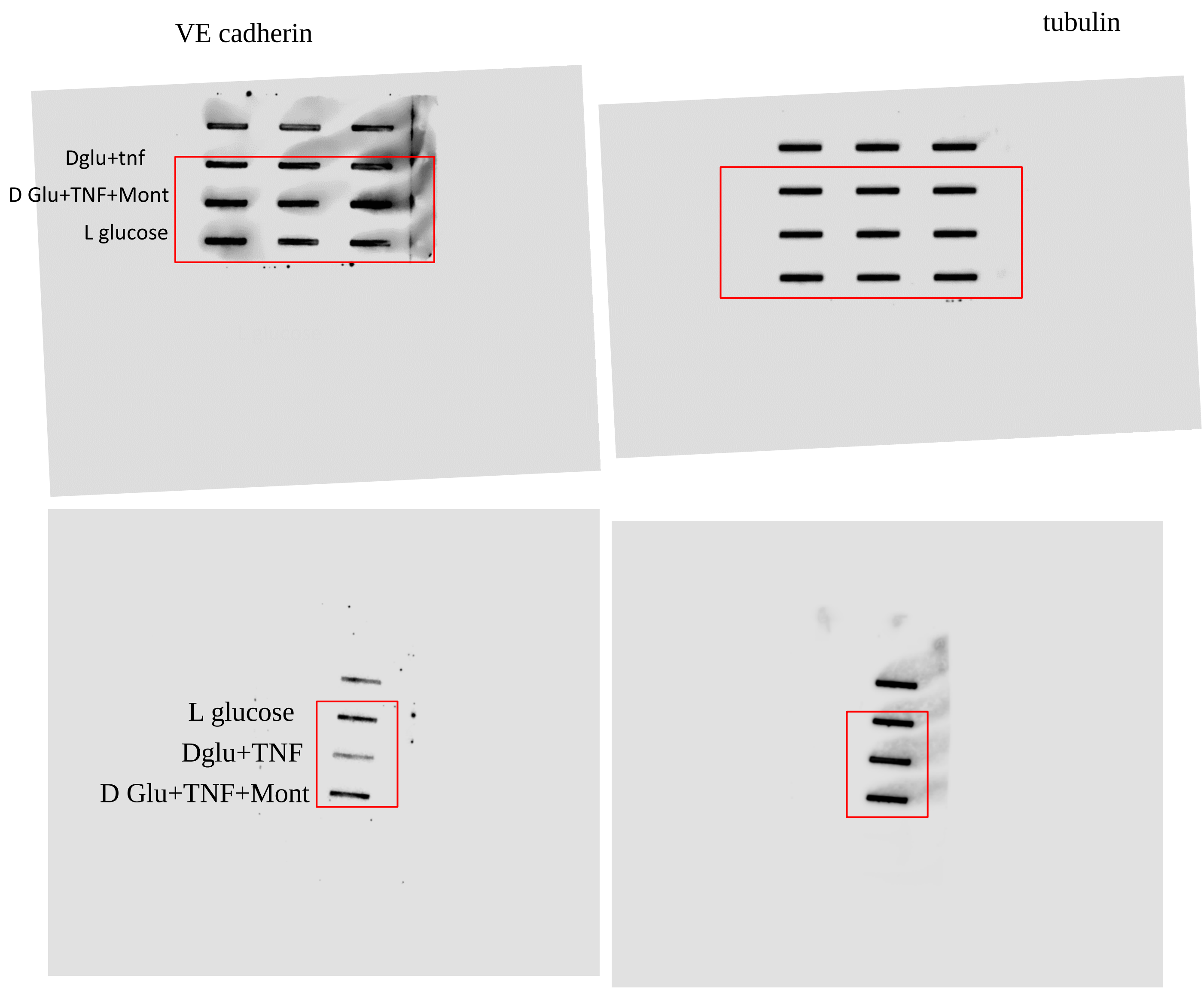
VE cadherin
tubulin
Dglu+tnf
D Glu+TNF+Mont
L glucose
L glucose
Dglu+TNF
D Glu+TNF+Mont

Figure 3H

CYSLTR (2h,6h,24h) Comparison blots

CYSLTR1

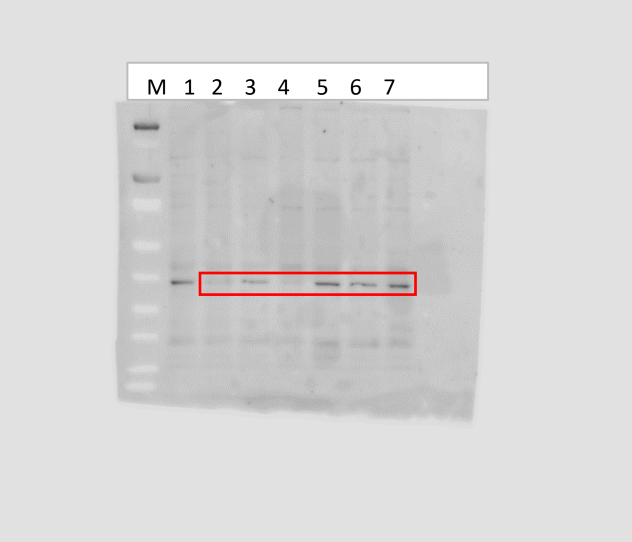

tubulin

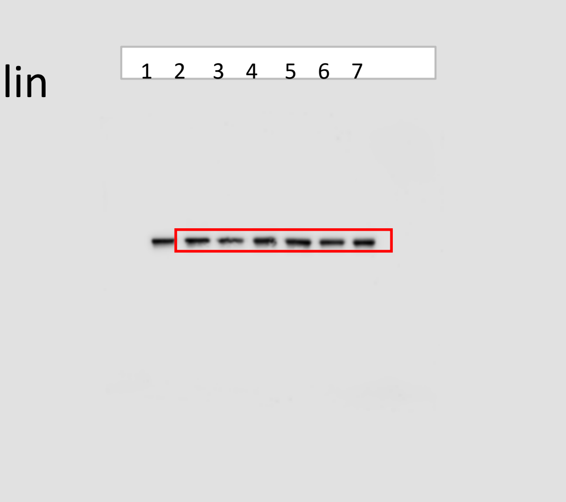

M: protein ladder

Lane1:DG 6h

Lane2:LG 2h

Lane3:LG 6h

Lane4:LG 24 h

Lane5: DG+TNF 2h

Lane6:DG+TNF 6h

Lane7:DG+TNF 24h

CYSLTR1

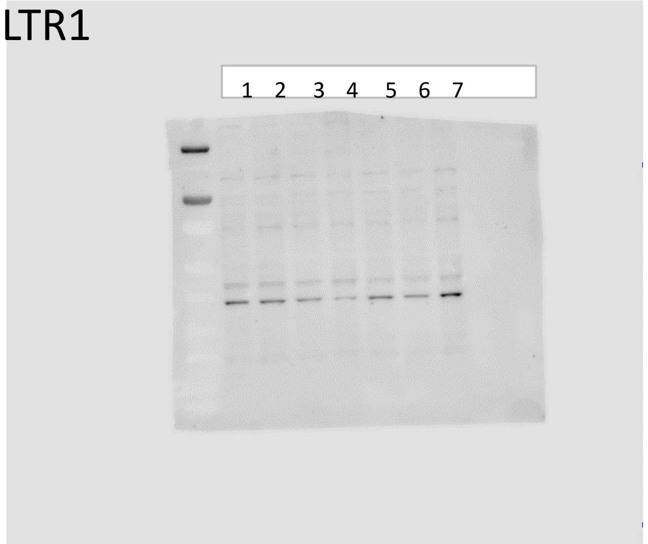

tubulin

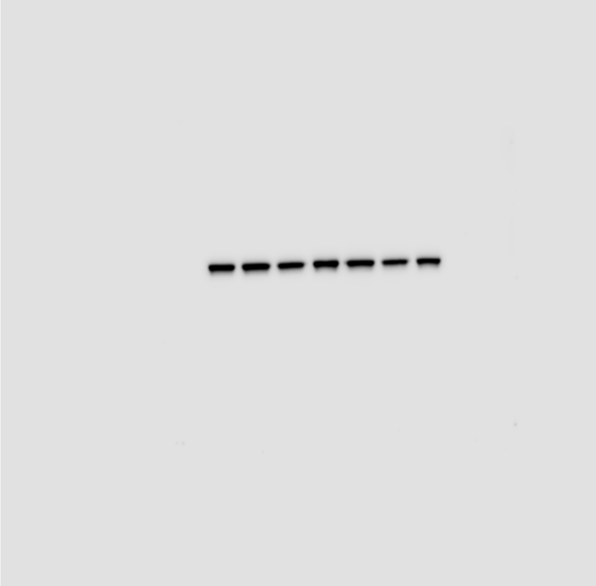

Figure 4B

Abcam cysltr1
HRE 6h treatment

P62 HRE 6h
treatment

Tubulin 6h
treatment

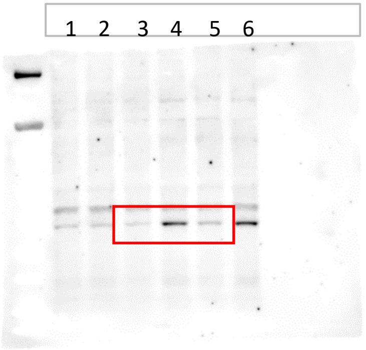

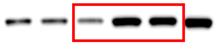

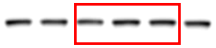

Lane1: Unstimulated cells

Lane2: Mannitol 30mM

Lane3: LG 30mM

Lane4:DG+TNF

Lane5:DG+TNF+MON5

Lane6:DG+TNF+MON2.5

Figure 4D

Transfection EXp1

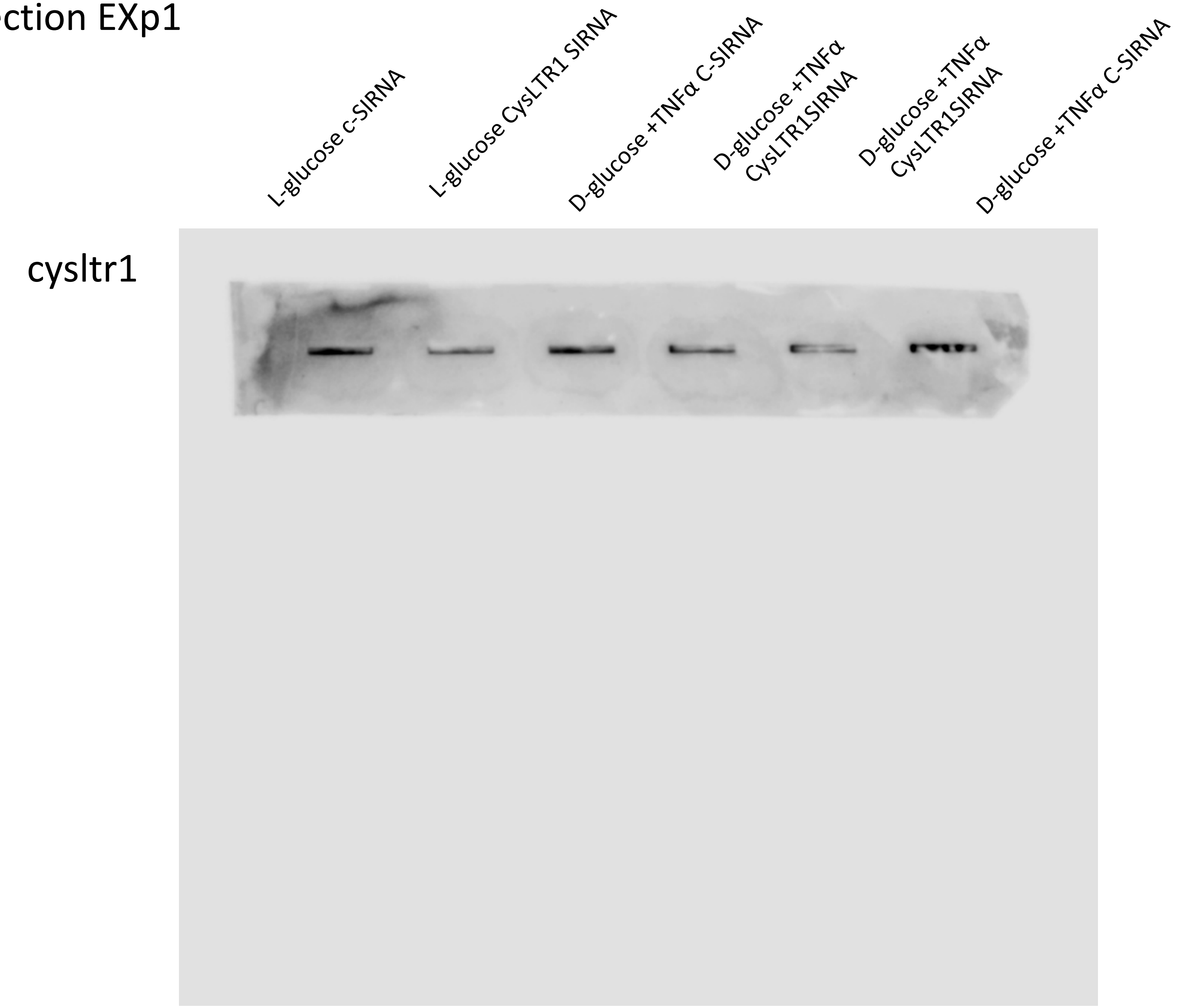

Figure 5B

Transfection EXp1

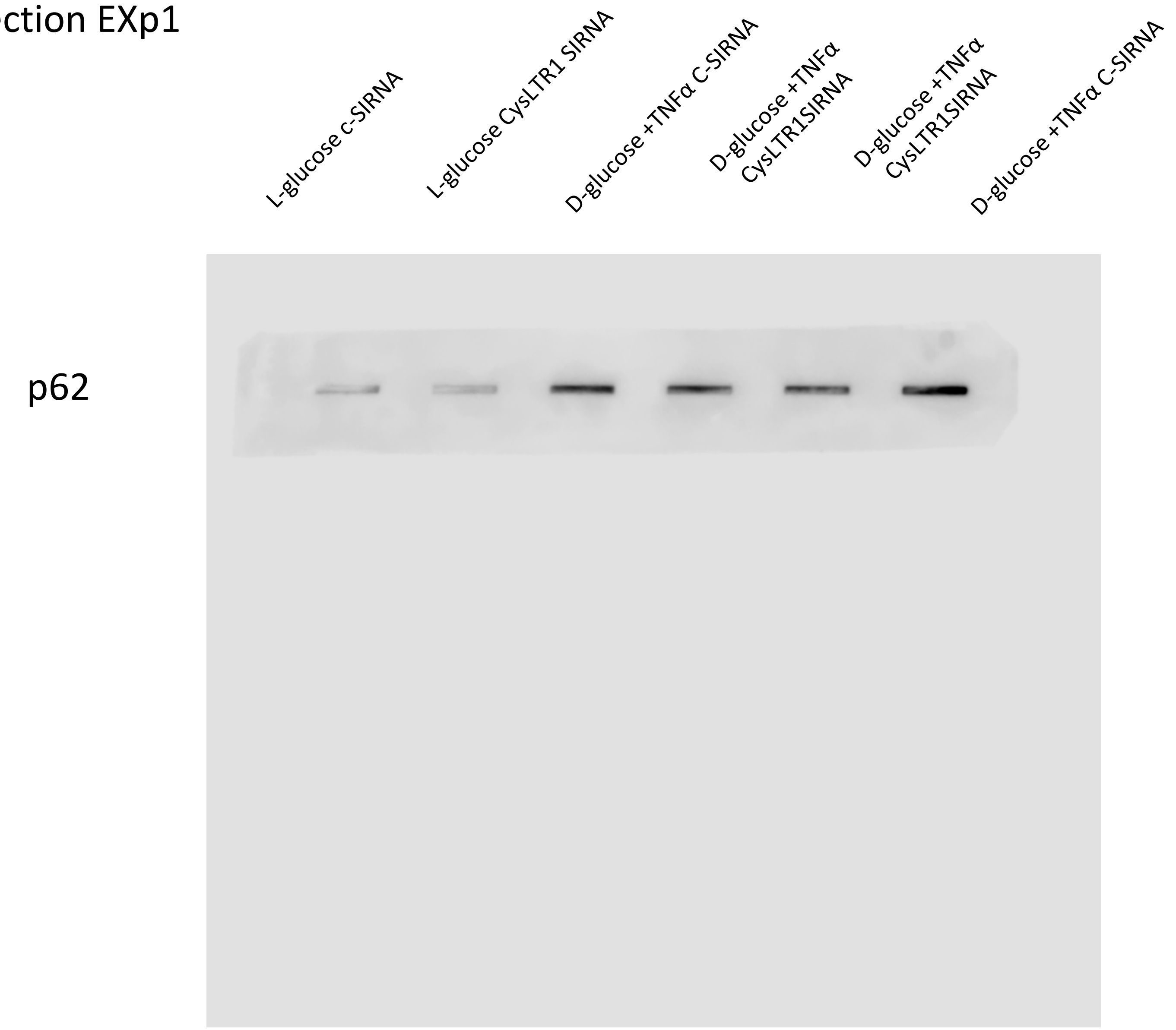

Figure 5C

Transfection EXp2

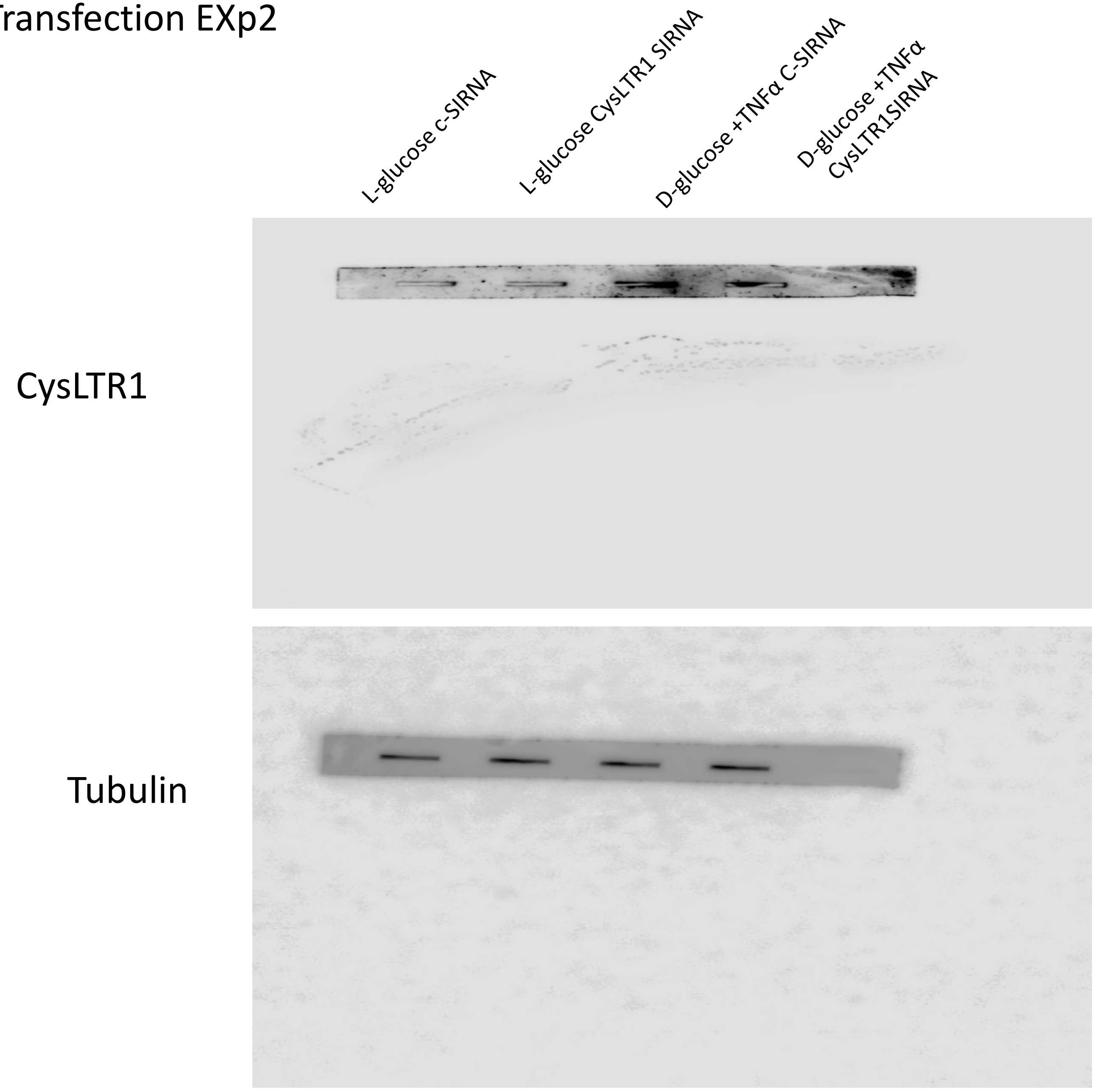

Figure 5B

Transfection EXp2

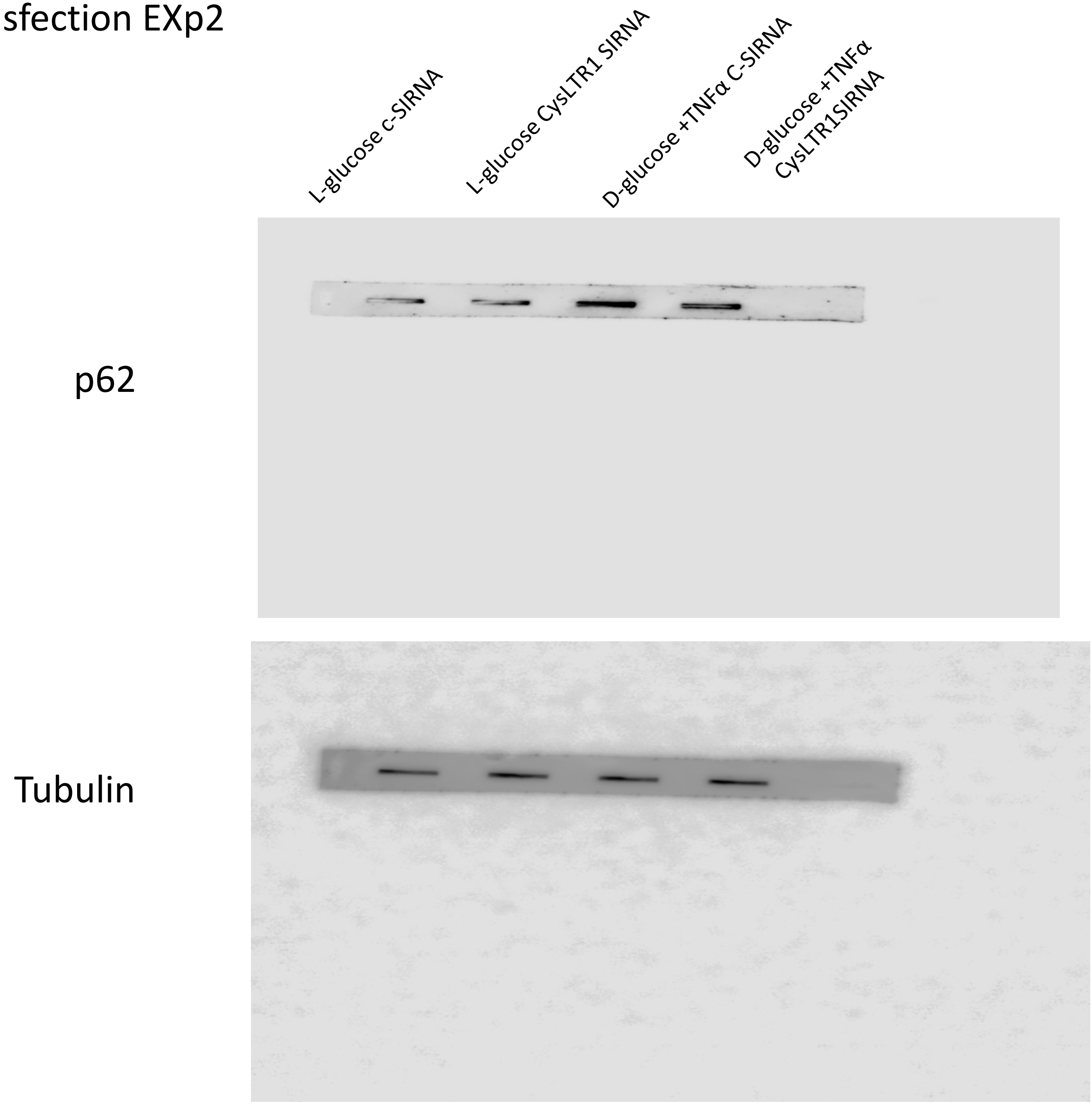

Figure 5C

Transfection EXp2

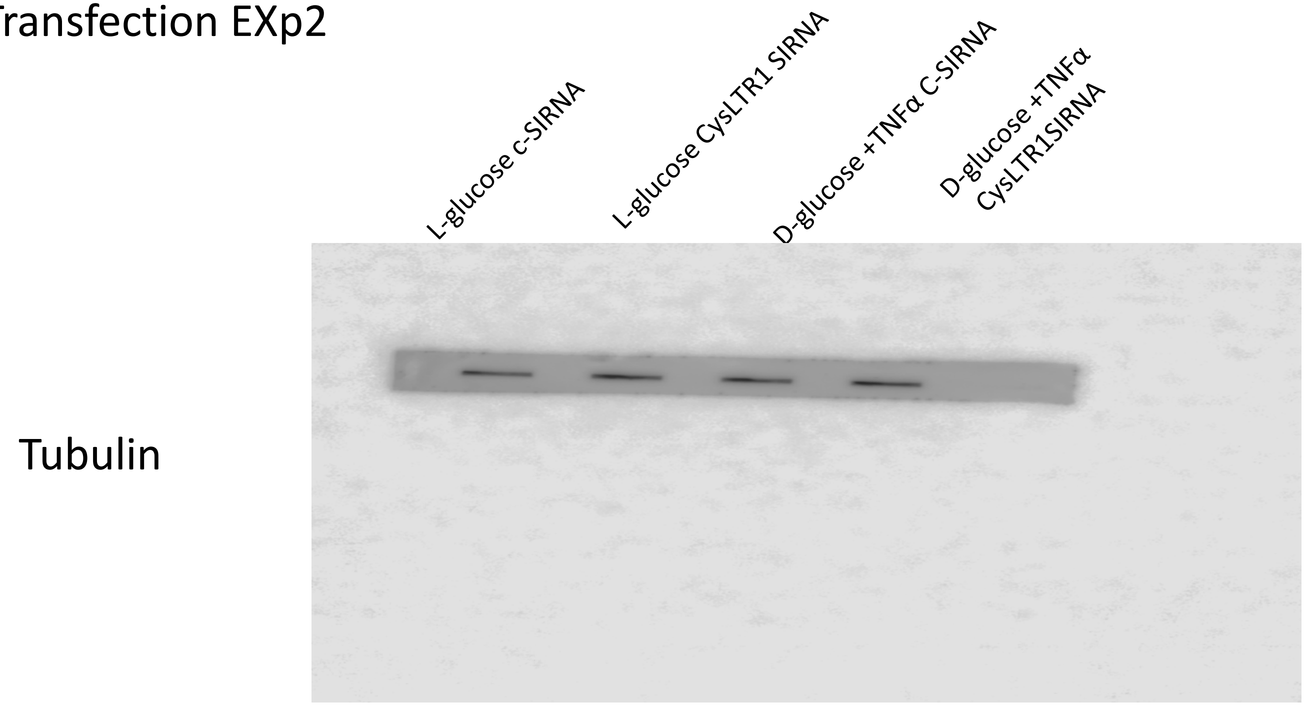

Figure 5

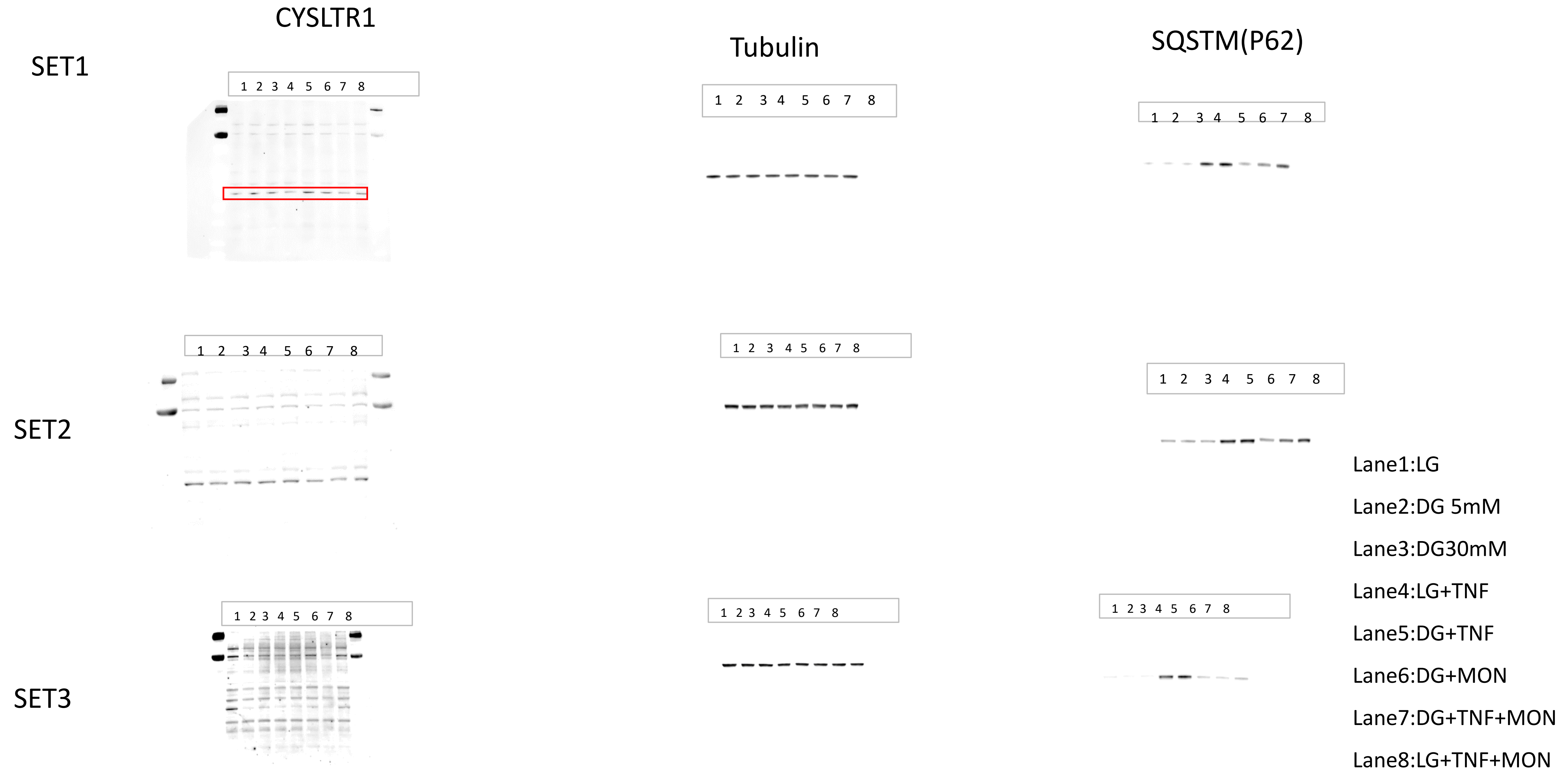

Supplemental Figure 1
